# Reaching task performance is associated to neuromuscular junction adaptations in rats with induced diabetes mellitus

**DOI:** 10.1590/1414-431X20208763

**Published:** 2020-06-03

**Authors:** Y.C. Estrada-Bonilla, P.A.T.S. Castro, G.L.F. Luna, A.B.A. Souza, G.S. Santos, T.F. Salvini, A.M.O. Leal, T.L. Russo

**Affiliations:** 1 Departamento de Fisioterapia, Universidade Federal de São Carlos, São Carlos, SP, Brasil; 2 Body, Subject and Education Research Group, Universidad Santo Tomás de Aquino, Bogotá, D.C., Colombia; 3 Departamento de Medicina, Universidade Federal de São Carlos, São Carlos, SP, Brasil

**Keywords:** Motor endplate morphometry, Neuromuscular junction morphometry, Diabetic myopathy, Motor performance, Reaching task performance, Muscular atrophy

## Abstract

Upper limb performance is affected by diabetes mellitus (DM). Neuromuscular junction (NMJ) is a key structure to understand the relationship between performance and morphology in DM. The aim of the study was to analyze NMJ plasticity due to DM in an animal model and its relationship with the function of forelimbs in rats. Twelve Wistar rats were divided into control (C) and DM groups. Animals were trained to perform a grasping task, following procedures of habituation, shaping, and reaching task. DM was induced using streptozotocin. Forelimb neuromuscular performance for dexterity was evaluated one day before DM induction and five weeks following induction. After that, biceps, triceps, and finger flexors and extensors were removed. Connective tissue and muscle fiber cross-sectional area (CSA) were measured. NMJ was assessed by its morphometric characteristics (area, perimeter, and maximum diameter), using ImageJ software. Motor performance analyses were made using single pellet retrieval task performance test. Student’s *t*-test was used for comparisons between groups. A significant decrease in all NMJ morphometric parameters was observed in the DM group compared with the C group. Results showed that DM generated NMJ retraction in muscles involved in a reaching task. These alterations are related to signs of muscular atrophy and to poor reaching task performance. In conclusion, induced DM caused NMJ retraction and muscular atrophy in muscles involved in reaching task performance. Induced DM caused significantly lower motor performance, especially in the final moments of evaluation, when DM compromised the tropism of the muscular tissue.

## Introduction

Diabetes mellitus (DM) is defined as a metabolic disease that causes hyperglycemia due to alterations in the release and/or function of insulin. According to the World Health Organization, an estimated 422 million adults were living with DM in 2014 around the world. In addition, prevalence has risen faster especially in low- and middle-income countries, negatively impacting health-care systems (1). Regarding functionality and independence in daily life activities, not only walking problems (2), but also deficiencies regarding reaching and manipulation are recurrent impairments experienced by diabetic people (3).

Understanding the link between neuromuscular performance and DM is an important topic for rehabilitation. Diabetic myopathy has been considered a common complication of DM, and it is associated with the failure to preserve muscle mass (atrophy), increase of fibrosis (4), and muscle weakness (5). Furthermore, retractions of neuromuscular junction (NMJ) and electrophysiological changes can also support muscle dysfunction (6–8) and early signs of diabetic neuropathy (9). On the other hand, NMJ alterations due to DM and neuromuscular performance are not totally understood. For example, changes in neuromuscular transmission of hindlimb muscles are not related to rotarod performance, that is, when the animal is placed on a rotating roller, walking at different speeds or at continuous acceleration, and the time latency is recorded when the animal falls on the rotarod device in DM mice model (8).

Much attention has been given to lower limb impairments due to DM in the literature, and less to upper limb performance. Reaching and grasping tasks are key movements for upper limb function and require dexterity (3). Studies conducted by Fortes et al. (9,10) and Thomson et al. (11,12) mention that smaller muscles, such as the muscles that move the fingers, elbows, and shoulders, which are related to the execution of manipulative tasks because they are constituted in a higher proportion by type II fibers, would be more susceptible to enter into cellular adaptations tending to atrophy. This is a response to stimuli that provoke this type of response (like DM), compromising motor performance by involving elements not only of a muscular order, but also of a neuromuscular order (NMJ and motor units). In addition, considering that movements are preserved among species (14), translational studies can bring important information about early signs of DM regarding function. Therefore, the present study investigated NMJ plasticity in a DM animal model and its associated function with forelimbs of rats. We hypothesized that NMJ retractions due to induced DM might be correlated to a worse reaching performance in rats.

## Material and Methods

The study was conducted according to the international standards of animal experimentation following the approval of the Ethics Committee regarding the use of animals (ECUA) from Federal University of Sao Carlos (UFSCar), code ECUA No. 4364131216/2017. The experiment followed the ARRIVE guideline recommendations and is in accordance with the National Institutes of Health guide for the care and use of laboratory animals.

### Animals and experimental design

Twelve 3-month-old male Wistar rats were pair-housed in cages in the Department of Biotech Physical Therapy at the UFSCar. A 12-h light/dark cycle was carried out with water access *ad libitum*. Animals were handled daily 2 to 3 weeks prior to the experiment, and all behavioral procedures were carried out in the same room. Prior to the start of behavioral procedures, animals were placed on a scheduled feeding of 15 g of rat chow pellets given once per day (to ensure that the animals did not lose weight during the training phase of the reaching task), according to what was determined in the protocol of habituation and shaping described by Jones (14) and Allred and Jones (15 [Bibr B16]). Weight was monitored throughout the study (2 weeks before DM induction, 1 day before DM induction, 1 week after DM induction, and 5 weeks after DM induction) using a digital scale (Toledo, USA) with a sensitivity of 0.01 g. In the procedure for measuring weight, each animal was introduced into a plastic container, subtracting the weight of the container from the result. To perform this subtraction, the “tare” function of the scale was used.

Animals were left in the training chamber (as described in the habituation and shaping section below) for 2 consecutive days (habituation period), then between 2 to 8 days more during procedures for shaping on the single-pellet retrieval task to determine forelimb dominance. After that, animals were trained in the retrieval pellet task for 10 days. After training, at 1 day pre-DM induction, animals underwent measurement of forelimb functional test (single-pellet retrieval test), and then, 24 h later, DM was induced (DM group, n=6). One day before euthanasia, animals performed a second test for measurement of forelimb function. Animals were euthanized 5 weeks after DM induction. Control (C) animals (n=6) performed the same procedures, with the exception of DM induction. The number of animals were determined considering the NMJ area variable as the main outcome.

### Habituation and shaping

All animals underwent a habituation period (20 min/day) for 2 consecutive days in a Plexiglas reaching chamber (30 cm long, 35 cm high, 15 cm wide) with a tall narrow window (1 cm wide and 3 cm high) in the center of the 15-cm side wall. For shaping, animals were placed in the same reaching chamber for 20 min. Animals reached with a forelimb through the small window for cereal (Froot Loops^®^), which was placed in front of a block, approximately 3 cm in height. The wells were centered with the left and right edges of the window at a distance of 1 cm from the window. A small Plexiglas rod of approximately 2 mm in diameter was adhered to the base of the reaching window and created a barrier that prevented animals from scraping the pellets into the chamber and also reduced attempts to use the tongue to retrieve pellets. When 20 consecutive reach attempts were performed with one limb during a 20-min session, this limb was identified as the preferred limb. Once animals reached this criterion, pre-operative shaping was ceased (14,15).

### Training in the single-pellet retrieval task

Training in the single pellet-retrieval task was carried out in a training chamber (14) (Supplementary Figure S1). During training, a Plexiglas wall was inserted into the reaching chamber ipsilateral to the animal's trained limb and cereal pellets were placed so that the animal could catch the pellet with the dominant forelimb. This wall effectively forced the animals to use the forelimb chosen by the experimenter for the reaching task. In the initial design of the apparatus, the inner chamber wall was placed at a distance of 1.5 cm from the reaching window.

The training with the preferred limb was carried out in all groups 10 days before the assessment of forelimb asymmetry and reach performance by the single-pellet retrieval tests. Animals were trained for 30 trials or a cutoff time (20 min), whichever came first (14). A reaching trial consisted of: a) if the animal either successfully grabbed the pellet and brought it directly to its mouth (success), b) dropped the pellet before bringing it to its mouth, or c) failed to grasp the pellet after five reaches or knocking the pellet out of its well.

At the end of each reaching trial, a pellet was dropped into either the front or the back of the reaching chamber to “re-set” the animals and so a new pellet was placed into its appropriate well. After diabetes induction (generating DM animal model), the DM and C groups underwent a new training set, with the same aforementioned characteristics. These periods of training were carried out until completing 2 consecutive weeks (14). After performing the second functional forelimb test, reaching task training was no longer carried out.

### Reaching task (single-pellet retrieval task)

Testing in the single-pellet retrieval task (or reaching task) was carried out one day before DM induction and 5 weeks after (one day before euthanasia). Reaching performance was calculated by dividing the total number of successful reaches by the total number of reach attempts with the preferred limb [(total success/total reach attempts) ×100], which corresponded to the percentage of successful reaches. The test was performed for 20 min (15–17).

### Diabetes mellitus induction and glycemia evaluation

Animals of the DM group received 50 mg/kg body weight of streptozotocin (Sigma-Aldrich, USA) in a single intraperitoneal dose dissolved in sodium citrate buffer. Animals from C group received the same volume of sodium citrate buffer only, according to Fahim et al. (18) and Morrow (19). One week after DM induction, rats with a blood glucose level above 250 mg/dL were considered to be diabetic and were used in the experiments. Body mass and fasting glycemia were determined weekly. Blood samples were collected from the tail vein, and blood glucose levels were measured by Accu-Check glucose meter (Roche Diagnostic, USA).

### Muscle sample collection

Biceps, triceps, and finger flexor and extensor muscles from dominant forelimbs were isolated, removed, and weighed. Upon removal (by anatomically guided atlas dissection), muscles were maintained in a 9% saline solution on a petri dish. Subsequently, each muscle was placed separately on a piece of aluminum foil to be weighed on a precision scale (Kern Instruments, USA). The weight of the aluminum foil was subtracted from the total weight using the “tare” function of the scale. Then, each muscle was divided into two parts. The proximal fragment was immersed in a glutaraldehyde solution and then used for the nonspecific esterase technique (20). A distal fragment of each muscle was used for histological morphometry, fixed on isopentane, pre-cooled in liquid nitrogen, stored at -80°C, and then used to measure both the cross-sectional area (CSA) of the muscle fibers and the percentage of connective tissue.

### Muscle fiber CSA

Histologic serial cross-sections were obtained from the biceps, triceps, and finger flexors and extensors (from the dominant forelimb) in a cryostat microtome (Leica, CM1860, Germany). A histologic cross-section (10 µm) stained with toluidine blue was selected to measure the CSA under a light microscope (Axiovision 3.0.6 SP4, Carl Zeiss, Germany) using morphometric analysis software (ImageJ software, version 1.43u, National Institutes of Health, USA). The CSA of each muscle was obtained by measuring 100 fibers located in the central region of the section. The percentage of connective tissue in each muscle was evaluated with the Image J software using a grid on a photo of each muscle to count the number of intersections of the grid that fell into the connective tissue. The percentage of points within the conjunctive period versus the number of points that the traced grid had was calculated (number of points or intersections within connective tissue/points or intersections of grid ×100).

### Neuromuscular junction analysis

The surface portions of biceps, triceps, flexors, and extensors (from dominant forelimbs) were sliced from the NMJ portion (containing the motor point), which was cut lengthwise into three or four slices. The resulting material was subjected to the nonspecific esterase technique (Lehrer technique) (20) to characterize the NMJ. Total area, total perimeter, and maximum diameter (21) were measured on 30 junctions with a light microscope (Axiovision 3.0.6 SP4, Carl Zeiss). The relative planar area (21,22) of each motor endplate was also calculated considering the ratio between total area/largest orthogonal axis of NMJ (21,22) previously evaluated. The measurements were analyzed with Image J software.

### Statistical analysis

All variables [total body weight, total glycemia, reaching performance (% of success), NMJ morphometry, and muscle components morphometry (CSA and % of connective tissue)] showed a normal and homogeneous distribution according to the Shapiro-Wilk and Levene tests, respectively. For variables of total body weight, total glycemia, NMJ morphometry, and histological morphometry of muscle tissue, comparison of averages of the pre-DM moment and 5 weeks post-DM induction within and between groups was carried out by the Student's *t*-test. For the motor performance, Student’s *t*-test was used to compare the variable % of success, between one day pre-DM induction and one day before euthanasia. Likewise, two types of correlations were established (with Tau Kendall's correlation coefficient): 1) between NMJ mean area, perimeter, and maximum diameter in each muscle from the DM group, with motor performance (% success) 5 weeks post-DM induction and 2) between the NMJ average area, perimeter, and maximum diameter in each evaluated muscle from the DM group with histological morphometric outcomes (CSA muscle fiber and % of connective tissue). Magnitude of correlations was based on the Munro’s classification [very low (0.15 to 0.24), low (0.25 to 0.49), moderate (0.50 to 0.69), high (0.70 to 0.89), and very high (0.90 to 1.00)] (23).

## Results

### Glycemia and total body weight

**Figure 1:**
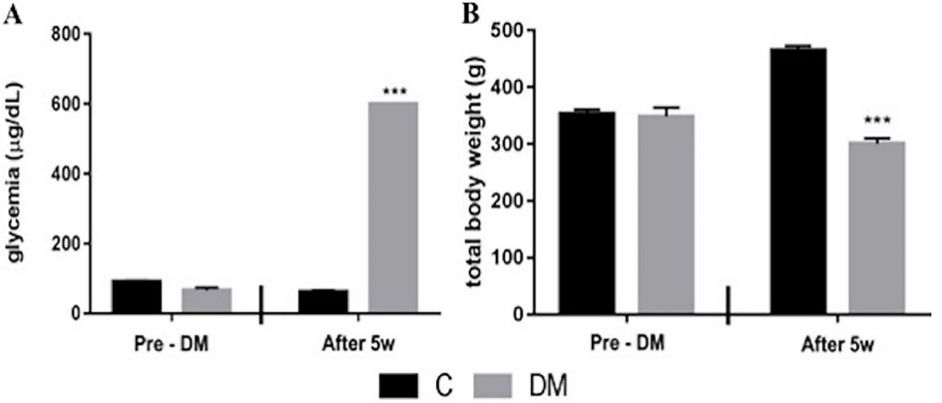
Mean values of total glycemia (**A**) and total body weight (**B**) in animals from control (C) and diabetes mellitus (DM) groups. Pre-DM: the moment before DM induction; After 5w: 5 weeks after DM induction. Data are reported as means±SE for n=6 animals per group ***P<0.01 compared to C (*t*-test).

The C and DM groups presented similar values of glycemia (p=0.198) and total body weight (p=0.922) at the pre-DM induction moment. The DM group had higher glycemia values (p=0.001) and lower total body weight (p=0.001) at the end of the experiment compared to the pre-DM induction group and also to the C group (both p=0.001, Figure 1).

### Motor performance

**Figure 2:**
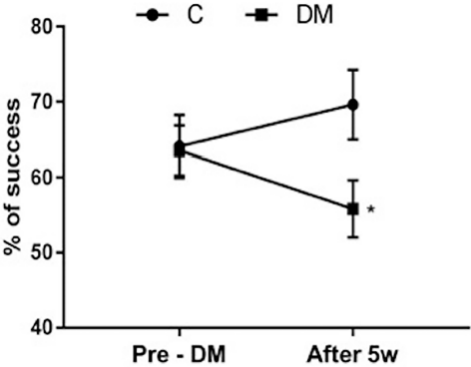
Percentage of success in motor performance in control (C) and diabetes mellitus (DM) groups. Pre-DM: the moment before DM induction; After 5w: 5 weeks after DM induction. Data are reported as means±SE for n=6 animals per group *P<0.05 compared to C (*t*-test).

At the pre-DM induction moment, both the DM and the C groups presented a similar percentage of reaching success (p=0.922; Figure 2). However, five weeks following DM induction, the DM group had a lower percentage of success compared to the control (p=0.04; Figure 2). Regarding the qualitative characteristics of evaluated motor performance, a general tendency was observed in the DM group who performed uncoordinated reaching task with mild dysmetria and tremors.

### Neuromuscular junction morphometric characteristics

**Figure 3:**
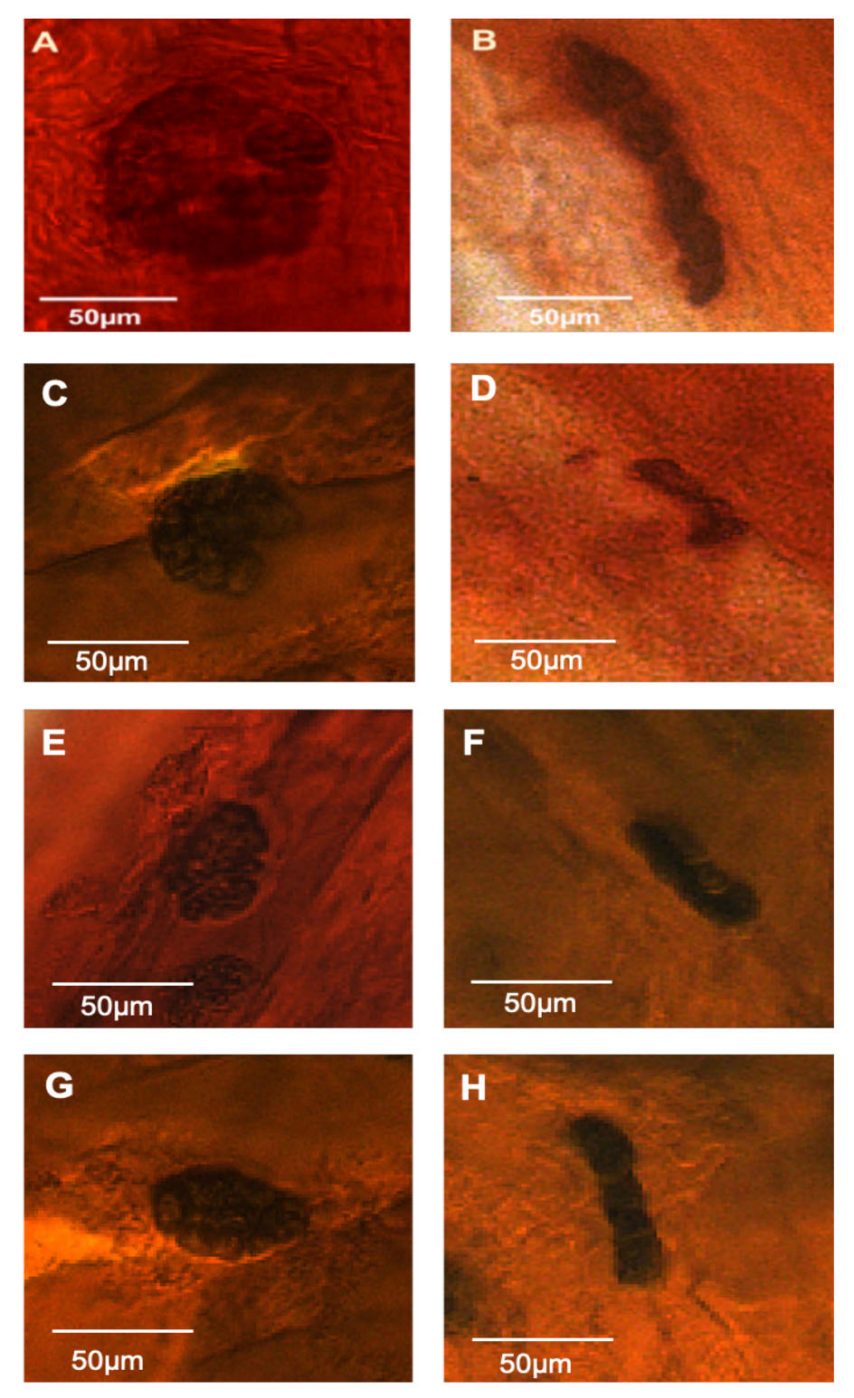
Comparison between neuromuscular junction morphology in biceps (**A**), triceps (**C**), finger flexor (**E**), and finger extensor (**G**) muscles of the control group and the same muscles in the diabetes mellitus group (**B**, **D**, **F**, **H**). Scale bar: 50 µm.

Animals from the DM group presented clear morphologic signs of decreased NMJs dispersions compared to individuals from group C. Furthermore, the NMJ presented more elongated and thinner shapes in DM animals compared to the typical “pretzel” appearance of control animals (Figure 3). Morphometric analyses showed a decrease in the NMJ total area, perimeter, maximum diameter, and relative planar in all investigated muscles compared to the control group (for more information see Figure 3 and Table 1).

**Table 1:** Morphometric characteristics of neuromuscular junction of biceps, triceps, finger flexor, and finger extensor muscles.

Neuromuscular junction	Biceps	Triceps	Finger flexor	Finger extensor
Total area (μm^2^)				
Control	243.5±8.10	275.5±9.67	217.9±7.71	205.1±6.88
Diabetes mellitus	96.1±5.75*	98.59±7.18*	99.21±6.65*	95.39±8.76*
Total perimeter (μm)				
Control	65.9±10.50	70.8±13.40	62.7±12.10	59.9±10
Diabetes mellitus	41.36±15.12*	43.60±21.14*	43.69±12.23*	42.05±13.59*
Maximum diameter (μm)				
Control	24.9±37.6	27.4±5.1	24.4±4.8	22.8±3.8
Diabetes mellitus	15.82±72.37*	17.06±1.01*	16.70±3.26*	16.34±4.64*
Relative planar area				
Control	8.89±1.7	9.0±1.6	7.9±1.30	8.6±1.2
Diabetes mellitus	6.05±0.165*	5.96±0.17*	6.16±0.41*	5.92±0.41*

Data are reported as means±SD for n=6 animals per group. *P<0.05 compared to Control (*t*-test).

### Muscle weight, muscle fiber CSA, and percentage of connective tissue

**Figure 4:**
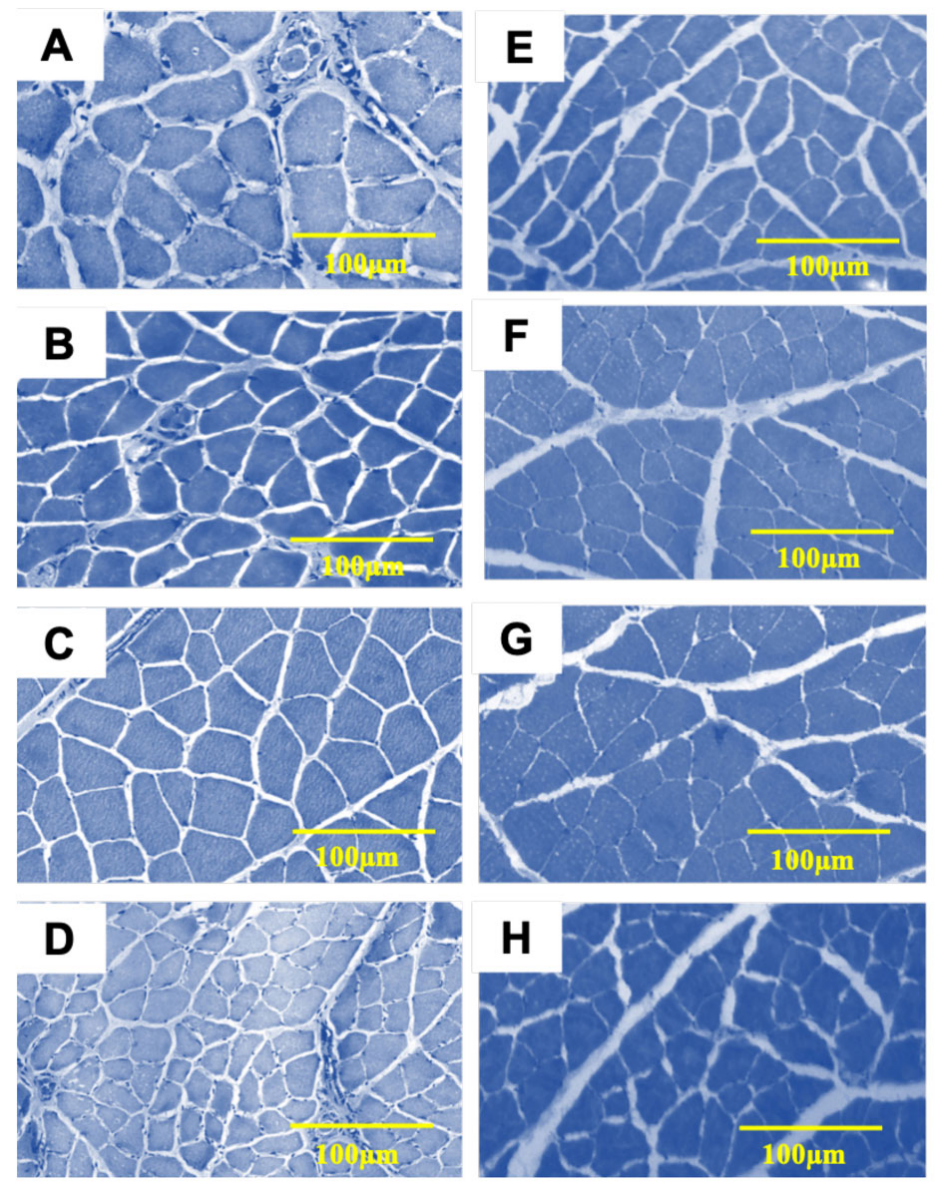
Comparison between triceps (**A**), biceps (**B**), finger flexor (**C**), and finger extensor (**D**) muscles of the control group and the same muscles in the diabetes mellitus group (**E**, **F**, **G**, **H**). Scale bar: 100 µm.

Only the finger flexor muscle weight decreased in DM rats compared to C rats (p=0.001). A reduction of muscle fiber CSA and an increase in percentage of connective tissue were observed in all muscles of the DM group compared to the C group (see Figure 4 and Table 2).

**Table 2:** Histological characteristics of biceps, triceps, finger flexor, and finger extensor muscles in diabetes mellitus (DM) and control (C) groups.

Muscles	Muscle fiber CSA (μm^2^)		Connective tissue (%)	
	C	DM	C	DM
Biceps	1996±1.89	1258±1.04*	6.3±0.6	40±5.7*
Triceps	2536±2.36	1797.7±1.4*	5.3±0.5	50.60±2.3*
Finger flexor	703±7.0	643.5±2.8*	5.7±0.9	42.72±2.3*
Finger extensor	841±5.1	524.1±2.0*	5.3±0.5	50.60±2.3*

Data are reported as means±SD for n=6 animals per group *P<0.05 compared to C (*t*-test). CSA: cross sectional area.

### Correlation between morphometric NMJ parameters and motor performance

A high positive correlation was observed for the NMJ mean area, perimeter, and maximum diameter of all muscles investigated with a percentage of success during the reaching task (e.g., the larger the area, perimeter, or maximum diameter, the better the motor performance) (Table 2).

### Correlation between morphometric NMJ parameters and muscle morphometric parameters

Moderate to high correlations were observed in the NMJ average area, perimeter, and maximum diameter versus the muscle fiber CSA and the percentage of connective tissue for analyzed muscles. Inverse correlations were observed between NMJ parameters and the percentage of connective tissue in all muscles. On the other hand, positive correlations were found between muscle fiber CSA and NMJ measurements for biceps, triceps, and finger flexors (Table 3).

**Table 3:** Correlations between morphometric characteristics of neuromuscular junction (NMJ) and motor performance in diabetes mellitus (DM) group and between morphometric characteristics of NMJ and morphometric characteristics of measured muscles (DM group).

Correlated variables	Tau Kendall's correlation coefficient			
	Biceps	Triceps	Finger flexor	Finger extensor
NMJ area *vs* % of success in motor reaching task test	0.914	0.958	0.926	0.876
NMJ perimeter *vs* % of success in motor reaching task test	0.878	0.845	0.930	0.944
NMJ maximum diameter *vs* % of success in motor reaching task test	0.909	0.965	0.955	0.850
NMJ area *vs* muscle fiber CSA	0.795	0.924	0.832	–0.949
NMJ maximum diameter *vs* muscle fiber CSA	0.899	0.840	0.842	–0.907
NMJ area *vs* % of connective tissue (in each muscle)	–0.940	0.871	0.808	–0.928
NMJ perimeter *vs* % of connective tissue (in each muscle)	–0.911	–0.880	–0.862	–0.907
NMJ maximum diameter *vs* % of connective tissue (in each muscle)	–0.943	0.871	–0.985	–0.928

Only correlations with a significant result are reported in this table. Number of animals: 6 per group. CSA: cross sectional area.

## Discussion

The present study showed for the first time that the DM animal model can induce NMJ retractions in muscles from dominant forelimbs in rats, and these modifications were correlated with reaching and grasping performance in rats. The decrease of the NMJ has already been reported regarding hindlimbs. Ramji et al. (13) showed that molecular alterations such as increased oxidative stress of the motor neurons is associated with the loss of distally supported terminals, reflecting the earliest aspects of neurodegeneration observed in DM. Either metabolic alterations in the resting membrane potential mechanisms of the axons, such as impaired Na^+^/K^+^-ATPase activity or slow motor nerve conduction velocity could also explain the deficits on muscle activation in early phases of type 1 DM. Furthermore, ultrastructural changes in diabetic NMJ such as disrupted T-tubules, decreased number of synaptic vesicles, and swollen mitochondria with irregular cristae are related to deficits in neuromuscular transmission and function (6,18,19).

Synthesis, transport, and reception of acetylcholine (ACh) are crucial for the structure and function of the components of the NMJ (24,25) and agrin, a glycoprotein that in conjunction with MuSK glycoprotein, is responsible for grouping or dispersing acetylcholine receptors (AChR) in muscle fibers in active sites of the NMJ. In DM, the AChRs are poorly grouped in the active zones of the NMJ (24–26). Additionally, the efficiency of the NMJ is also related to the number of crests with “pretzel” form (27-29). The present study described the loss of typical “pretzel” form of the NMJs in all muscles investigated, which suggested a decrease of crests or active zones of grouping of AChR.

More recently, it was demonstrated in animals that alpha and gamma neuron populations are decreased at 22 and 33 weeks of DM, respectively. In addition, the DM animal model can provoke muscle spindle denervation (30,31). Together, these studies might contribute to the understanding of the muscle weakness observed in DM. Reduced muscle strength as a complication of DM has been reported in humans as well. A decrease in concentric and isometric peak torques of knee and ankle muscles in diabetic individuals occurs even before the onset of diabetic neuropathy (32).

The literature shows that morphological modifications of NMJ or muscle tropism due to DM failed to show deficits on animal performance in functional tests, such as the rotarod test. It was assumed that compensatory mechanisms, such as axonal sprouting for reinnervation of denervated muscle fibers, occurred as a compensatory measure to maintain the function (7). On the other hand, Sabadine et al. (33) have recently found that DM impaired walking in rats. The present study challenged studies that investigate hindlimbs and showed that DM impaired reaching performance, applying the findings regarding animals with DM to clinical practice.

Regarding the effects of DM on upper limb performance, it has been described that grip strength is reduced in the diabetic population, reflecting general muscle weakness (34,35). Furthermore, it was demonstrated that DM individuals present an altered dynamical coordination of intrinsic muscles during precision gripping. Cederlund et al. (36) attributed these alterations to muscular remodeling, altered motor units firing patterns, and deficits on forward feedback mechanisms due to sensorial deficits (35). Other clinical aspects that may occur in patients with DM are Dupuytren's contracture and impaired vibrotactile sense in finger pulps, and a longer duration of DM was associated with more severe neuropathy and difficulties in activities of daily living (36).

Furthermore, results obtained in the present study showed that the evaluated muscle groups had signs of muscle atrophy associated with the development of DM. We investigated four main groups of muscles related to the movements of the forelimb, contrasting with previous studies that investigated one or two muscles of hindlimbs. The literature suggests that insulin-dependent DM generates structural and histological alterations of skeletal muscle tissue, recognized as diabetic myopathy. The most relevant changes are: a) reduction of the CSA of muscle fibers, b) shifts of myosin-heavy chains types (fiber I to II conversion), and c) conformational and organizational type changes of collagen in the extracellular matrix (ECM) of muscle tissue, such as an increase in the amount of connective tissue (fibrosis) (37–39).

The aforementioned studies also confirm that the characteristics of muscle changes in diabetic myopathy are directly due to the development of oxidative stress, which is characterized by the decrease of the reparative capacity of satellite cells, generating, in turn, an imbalance in the repair tissue process, a decrease of regulatory proteins of the tissue repair process (MyoD, myogenin, and JunD), as well as a decrease in the synthesis of local vasodilator agents (specifically, nitric oxide - NO). Similarly, it is also evident that in the presence of hyperglycemia there are important quantities of reactive oxygen species, which lead to the synthesis of metalloproteinases, thus explaining the degradation and/or catabolism of collagen in the ECM. Together, all the factors triggered by oxidative stress are responsible for the structural and histological muscular tissue changes (39).

The findings described above are complemented by studies conducted by Fortes et al. (9,10) and Marzuca-Nassr et al. (40) whose objectives were to observe the ability of a muscle to recover after being exposed to an atrophy triggering factor (such as DM in animal models of this disease). The previous statement is related to observed aspects such as the average decrease of the CSA, more specifically in muscles that, although having a mixed conformation, are predominantly formed by type II fibers, as is the case of the finger flexor and extensor muscles. In addition, the increase in the percentage of connective tissue is more marked in the aforementioned muscle groups as well as significantly lower morphometric characteristics of the NMJ, which were observed in all muscle groups analyzed, but in a much more marked way in finger flexors and extensors.

The fact that smaller muscles such as finger extensors and flexors, with such specific functions as the reaching task, are constituted mainly by type II fibers, may explain a lower recovery capacity following DM evolution, compensatively increasing the amount of connective tissue, trying to improve its function. However, in an acute phase of DM, the possible start of a diabetic myopathy 5 weeks post-induction can cause a decrease in motor performance in the execution of the reaching task.

This study had some limitations. For example, the absence of peripheral nerve investigation did not allow the quantification of the possible effects of diabetic neuropathy. In addition, molecular analyses could bring important insights regarding the pathophysiology of NMJ modifications in the induced DM model. However, in the first 5 weeks of induced DM, diabetic myopathy is more accentuated than neuropathy. This can be explained based on the aforementioned aspects, such as the metabolic conformation of the muscle according to its fiber types (9–12), as well as its number of motor units, which in turn relates to the functions of the muscle (23).

Normally, muscles constituted of glycolytic fibers in greater proportion are more easily affected than oxidative muscles. This can lead to a compensation process that is finalized with an increase of the synthesis of extracellular matrix proteins, to try to maintain muscle mass, with loss of function. Finally, this study showed important correlations between NMJ, muscle atrophy and fibrosis formation, and reaching task performance. More accurate evaluations of upper limb movements, particularly grasping or pinching, might indicate the first signs of neurodegeneration and myopathy linked to DM. In addition, specific tasks or strength training might be an interesting therapeutic strategy to avoid deleterious morphological and functional alterations.

In conclusion, the DM animal model seems to be a good model for studying early modifications in reaching tasks. DM induced NMJ retractions, muscle atrophy (with CSA decrease), and fibrosis (with connective tissue increase) in muscles of the forelimb, which are related to reaching tasks. These types of structural and physiological alterations can explain the alterations in motor performance, reflected in a modified execution of manipulative/reaching tasks. This situation, observed in DM animal models, could be similar to what occurs in the evolution of DM in humans, although the time for observing these changes varies.

## Supplementary Material

Click here to view [pdf].
